# Spatial and temporal epithelial ovarian cancer cell heterogeneity impacts Maraba virus oncolytic potential

**DOI:** 10.1186/s12885-017-3600-2

**Published:** 2017-08-30

**Authors:** Jessica G. Tong, Yudith Ramos Valdes, Milani Sivapragasam, John W. Barrett, John C. Bell, David Stojdl, Gabriel E. DiMattia, Trevor G. Shepherd

**Affiliations:** 1 Translational Ovarian Cancer Research Program, London, ON Canada; 2 Translational Head and Neck Cancer Research Program, London, ON Canada; 3 Department of Anatomy & Cell Biology, Western University, London, ON Canada; 4 Department of Biochemistry, Western University, London, ON Canada; 5 Department of Oncology, Western University, London, ON Canada; 6 Department of Obstetrics & Gynaecology, Western University, London, ON Canada; 7 Department of Medicine & Biochemistry, University of Ottawa, Ottawa, ON Canada; 8 Department of Microbiology and Immunology, University of Ottawa, Ottawa, ON Canada; 9 Department of Pediatrics, University of Ottawa, Ottawa, ON Canada; 10 London Regional Cancer Program, 790 Commissioners Road East, Room A4-836, London, ON N6A 4L6 Canada

**Keywords:** High-grade serous ovarian cancer, Tumour heterogeneity, Ascites, Oncolytic virus, Maraba virus, Resistance

## Abstract

**Background:**

Epithelial ovarian cancer exhibits extensive interpatient and intratumoral heterogeneity, which can hinder successful treatment strategies. Herein, we investigated the efficacy of an emerging oncolytic, Maraba virus (MRBV), in an in vitro model of ovarian tumour heterogeneity.

**Methods:**

Four ovarian high-grade serous cancer (HGSC) cell lines were isolated and established from a single patient at four points during disease progression. Limiting-dilution subcloning generated seven additional subclone lines to assess intratumoral heterogeneity. MRBV entry and oncolytic efficacy were assessed among all 11 cell lines. Low-density receptor (LDLR) expression, conditioned media treatments and co-cultures were performed to determine factors impacting MRBV oncolysis.

**Results:**

Temporal and intratumoral heterogeneity identified two subpopulations of cells: one that was highly sensitive to MRBV, and another set which exhibited 1000-fold reduced susceptibility to MRBV-mediated oncolysis. We explored both intracellular and extracellular mechanisms influencing sensitivity to MRBV and identified that LDLR can partially mediate MRBV infection. LDLR expression, however, was not the singular determinant of sensitivity to MRBV among the HGSC cell lines and subclones. We verified that there were no apparent extracellular factors, such as type I interferon responses, contributing to MRBV resistance. However, direct cell-cell contact by co-culture of MRBV-resistant subclones with sensitive cells restored virus infection and oncolytic killing of mixed population.

**Conclusions:**

Our data is the first to demonstrate differential efficacy of an oncolytic virus in the context of both spatial and temporal heterogeneity of HGSC cells and to evaluate whether it will constitute a barrier to effective viral oncolytic therapy.

**Electronic supplementary material:**

The online version of this article (10.1186/s12885-017-3600-2) contains supplementary material, which is available to authorized users.

## Background

Late-stage diagnosis of epithelial ovarian cancer (EOC) and acquisition of chemotherapeutic resistance in recurrent disease are the major contributors to poor patient prognosis [1, 2]. Debulking surgery either before or after adjuvant chemotherapy is the standard treatment for ovarian cancer patients with metastatic disease. Under this treatment regimen, EOC is still the most lethal gynaecologic malignancy in the developed world with a 5-year survival rate of less than a 30% [3]. Following chemotherapy, the selection and expansion of platinum-resistant EOC cells results in the recurrence of aggressive disease that is largely incurable with second-line treatment options. Chemoresistance, particularly in high-grade serous cancer (HGSC) of the ovary, the most common histotype of EOC, is fueled by profound genomic instability caused by DNA repair pathway deficiencies and universal loss of *TP53* which results in a high degree of intratumoral cellular heterogeneity [4–6]. As observed in many cancers, intratumoral heterogeneity generates a high degree of phenotypic variability which can manifest as differential responses to therapies. Thus, there is significant demand for more effective therapeutics that target disease heterogeneity more effectively, thereby increasing progression-free survival for these patients.

Cancer cells naturally gain survival- and growth-enhancing properties through the selection and expansion of specific clones within a tumour. In doing so, aggressive cancer cells may also lose many intracellular pathogen defense mechanisms while inducing immunosuppressive mechanisms. Oncolytic virotherapy exploits these defects in intracellular defense to selectively replicate in malignant cells [7]. Additional changes in the tumour microenvironment, such as decreased immune surveillance, also enhance virus targeting of cancers. For example, mutations in interferon (IFN) and in other proteins in this signaling pathway are frequently seen in cancer cells as they are major drivers of anti-tumour immunity [8]. However, type I IFNs are also key antiviral signaling molecules found in all somatic cells thereby making cancer cells selectively infected and killed by oncolytic viruses [9]. Many rhadbdoviruses, including Maraba virus (MRBV), represent promising oncolytic viral vectors because of their susceptibility to IFN signaling as well as innate and adaptive immune responses making these viruses relatively non-pathogenic in healthy humans. Thus, tumours that are deficient in immunosurveillance pathways have increased susceptibility to these viruses. Currently, a construct of MRBV armed with a tumour-associated antigen, MAGE A3 is being evaluated in a phase I/II clinical trial in conjunction with adenovirus-MAGE A3 to investigate their immunostimulatory activity and oncolytic potential (clinicaltrials.gov identifier: NCT02285816).

In a previous cross-comparison of three oncolytic viruses, we observed potent oncolytic effects of MRBV in several EOC cell lines [10]. Infections of EOC cell lines cultured as adherent cells and three-dimensional spheroids in suspension revealed that MRBV was the most potent at inducing oncolysis. Furthermore, we identified the low-density lipoprotein receptor (LDLR) and its family members as partial mediators of MRBV entry that may be used to predetermine MRBV oncolysis of cancer cells. However, the potential for resistance to MRBV treatment has yet to be determined in a heterogeneous EOC model. Herein, our objective was to examine the efficacy of MRBV infection and oncolytic killing in the context of temporal and spatial heterogeneity of malignant EOC cells from a patient with recurrent disease. Direct analysis of multiple isolates from this patient with metastatic HGSC of the ovary may provide evidence for intratumoral heterogeneity impacting MRBV oncolytic efficacy. Moreover, it is unclear whether temporal changes in a tumour cell population, particularly after chemotherapy, may cause molecular and cellular changes that affect MRBV infection and oncolysis. Thus, we hypothesized that the high degree of tumour cell heterogeneity in ovarian HGSC would yield differential MRBV oncolytic efficacy and potential resistance mechanisms.

## Methods

### Cell culture

**Fig. 1:**
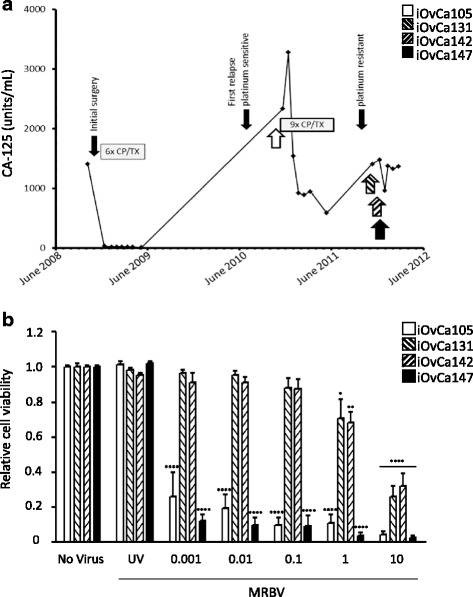
Temporal tumour cell biology impacts MRBV oncolytic efficacy in vitro. **a**. Serum CA-125 concentration (units per mL) for the patient from whom ascites samples were collected to derive new cell lines from October 2008 to March 2012 comprising the complete clinical course of her disease. iOvCA105, iOvCa131, iOvCa142, and iOvCa147 samples were derived from multiple ascites isolated upon first relapse and over a 14-month period (*upward arrows*). iOvCa105 cells were isolated October 2010 after first relapse with platinum-sensitive disease. iOvCa131, iOvCa142, and iOvCa147 were collected in close succession one year later between October 2011 and December 2011 upon second recurrence and acquisition of platinum resistance. **b**. Cells from all four cell lines were seeded at 10,000 cells per well of a 96-well plate and infected with different doses of MRBV as indicated, or UV-inactivated MRBV as a control. Cells were assayed for viability using CellTiter-Glo® reagent at 72 h post-infection. (*, *p* < 0.05; **, *p* < 0.01; ***, *p* < 0.001; ****, *p* < 0.0001, as determined by one-way ANOVA and Dunnett’s *posthoc* test)

The patient in this study initially presented with stage IIIC disease and was managed by surgical debulking followed by six cycles of carboplatin and paclitaxel combination chemotherapy. Histopathological assessment concluded that this patient’s malignancy displayed a mixed tumour morphology consisting of 70% serous and 30% clear cell adenocarcinoma. Upon disease recurrence, ascites fluid was collected on four different occasions (Fig. 1a) by paracentesis to initiate primary cell cultures as described previously [11]. To establish early-passage cell lines iOvCa105, iOvCa131, iOvCa142, and iOvCa147, malignant cells were propagated after removal of non-cancer cells by differential trypsinization from the mixed ascites-derived cultures. The iOvCa147 line was used in limiting-dilution subculturing with each well of two 96-well cluster dishes seeded at 0.3 cells per well. Subclones were expanded from single cells to generate all seven lines used in this study: iOvCa147-B3, −C8, −E2, −F5, −F8, −G4, and -G7. All cell lines and subclones were subjected to short-tandem repeat (STR) analysis, which verified that they originated from the single source. All cell lines were cultured continuously in Dulbecco’s Modified Eagle medium/Ham’s F12 (Wisent) supplemented with 10% FBS (Wisent). Cells were grown in a 37 °C humidified atmosphere of 95% air and 5% CO_2_. Adherent cells were maintained on tissue culture-treated polystyrene (Sarstedt, Newton, NC). Spheroids were maintained on Ultra-Low Attachment (ULA®) cultureware (Corning, Corning, NY), which is coated with a hydrophilic, neutrally charged hydrogel to prevent cell attachment. All patient-derived cells were used in accordance with The University of Western Ontario Human Research Ethics Board approved protocol (UWO HSREB 12668E).

### Virus production

Vero cells were infected with MRBV at multiplicity-of-infection (MOI) 0.01 [9]. Twenty hours after infection, supernatant was collected and virus was purified using a 0.2-μm filter [10]. MRBV MG1 mutant strain expressing GFP used in these experiments has been described previously [9].

### Virus infection of EOC cells

Primary EOC cells were seeded at 10,000 cells per well of a 96-well plate and were infected the following day at MOI 0.001, 0.01, 0.1, 1, and 10. The appropriate UV-inactivated virus at MOI 10 or no virus (mock-infected) were used as controls. Seventy-two hours after infection, viability was assayed using CellTiter-Glo® Luminescent Cell Viability Assay (Promega, Madison, WI). For infection of EOC spheroids, cells were seeded at 50,000 cells per well of a 24-well ULA cluster plate (Corning, Corning, NY) and spheroids were allowed to form over 72 h. Spheroids were then infected at MOI 0.01, 0.1, 1, and 10 using the same controls as described for adherent cell infections. Phase contrast and fluorescent images of infected cells and spheroids were captured during each experiment using a Leica DMI 4000B inverted microscope.

### Virus entry quantitation

iOvCa147-F8 and iOvCa147-G4 cells were infected with MRBV at an MOI of 0.1 at 4 °C to allow synchronous virus adsorption to the cells. After 1 h, supernatant containing uninfected virus was removed and titrated on Vero cells. Agarose overlay and plaque assay was performed to determine virus concentration through limiting dilutions. Virus infection containing no cells was performed as a negative control to normalize total MRBV concentration collected at 0% infection.

### *LDLR* knockdown

iOvCa147-F8 and iOvCa147-E2 cells were seeded at 20,000 cells per well of 48-well plates and transfected 16 h after seeding with s*iLDLR* SMARTPool RNA or with siNT (non-targeting control siRNA) using DharmaFECT1 transfection reagent (Dharmacon). At 48 h post-transfection, cells were used for infection experiments. The LDLR-related family member inhibitor, receptor-associated protein (RAP), was used at a concentration of 100 nM for 1 h [12] DMSO served as a vehicle control. After incubation, MRBV was added and virus entry and viability were assessed as described above.

### Media swapping experiments

#### Media swap pre-infection

Cells were seeded at 10,000 cells per well of a 96-well plate. Sixteen hours post seeding, conditioned media from both iOvCa147-F8 and iOvCa147-G4 was either replaced with fresh media, swapped between the two clones, or left unchanged. Cells were then infected at an MOI of 0.1 for 1 h and viability was assessed 48 h after infection by CellTiter-Glo®.

#### Media swap post-infection

Cells were seeded at 10,000 cells per well of a 96-well plate. Sixteen hours post-seeding, cells were infected with MRBV at MOI 0.1 for 1 h followed by media change. At 12 h, fresh media was either replaced, conditioned media was swapped between iOvCa147-F8 and iOvCa147-G4 subclone cells, or were left unchanged. CellTiter-Glo® assays were performed for cell viability 48 h after infection.

### Quantitative RT-PCR

iOvCa147-F8, iOvCa147-G4, and iOvCa147-B3 clones were seeded at 500,000 cells per well of a 6-well plate. Sixteen hours post-seeding, cells were infected with MRBV at an MOI of 1 or UV-inactivated MRBV at an MOI of 1 for 6 h. The A549 lung carcinoma cell line was used as a positive control for an intact type I IFN response [13]. Total RNA was isolated from both non-infected and infected cells using Qiagen RNeasy Mini Kit (Qiagen, Valencia, CA). Purified RNA was quantified using an ND-1000 spectrophotometer (NanoDrop technologies, Wilmington, DE). Reverse transcription was performed using total RNA isolated and Superscript II reverse transcriptase (Invitrogen) as per manufacturer’s instructions. PCR reactions were carried out using Brilliant SYBR Green QPCR Master Mix (Agilent Technologies/Stratagene) and a Stratagene Mx3000P machine with data exported to Microsoft Excel for analysis. *IFNβ1* and *GAPDH* primers were used as previously described [13]. *GAPDH* served as an internal control for RNA input and quantification was performed using the ∆∆Ct method [14].

### Co-culture experiments

Co-cultures of iOvCa147-F8 and iOvCa147-G4 cells were seeded in 24-well plates at a total of 100,000 cells per well. Wells containing only 100% iOvCa147-F8 or iOvCa147-G4 cells were used as normalization controls for MRBV effect on viability (sensitive and resistant, respectively). An increasing proportion of iOvCa147-F8 cells were titrated into the iOvCa147-G4 co-culture (G4:F8 ratio: 98:2, 90:2, 75:25, 50:50, and 25:75) with total number of cells consistent at 100,000 cells per well. Sixteen hours post-seeding, cells were infected with MRBV at an MOI of 0.05 and viability was measured 48 h post-infection using CellTiter-Glo®.

#### Cell tracker dye co-culture images

Confluent 10-cm plates of iOvCa147-F8 cells were stained with Molecular Probes™ Lipophilic Tracer DiI at 1:500 dilutions (ThermoFisher Scientific, Waltham, MA). Confluent 10-cm plates of iOvCa147-G4 cells were stained with CellTracker™ Blue CMAC Dye at a 1:500 dilution (ThermoFisher Scientific, Waltham, MA). Cells were stained for 1 h and subsequently seeded and infected as described above. Fluorescent images (GFP, DiI, and CMAC) were captured at 24 h post-infection using a Leica DMI 4000B inverted microscope.

### Immunoblotting

Cell lysates were generated using a modified radioimmunoprecipitation assay (RIPA) buffer [50 mM HEPES pH 7.4, 150 mM NaCl, 10% glycerol, 1.5 mM MgCl_2_, 1 nM ethylene glycol tetraacetic acid, 1 nM sodium orthovanadate, 10 mM sodium pyrophosphate, 10 mM sodium fluoride, 1% Triton-X-100, 1% sodium deoxycholate, 0.1% sodium dodecyl sulfate, 1 mM phenylmethylsulfonyl fluoride, 1× protease inhibitor cocktail (Roche, Laval, QC)] as described previously [10]. Lysates were incubated on ice for 20 min and vortexed to ensure complete lysis prior to centrifugation. Protein concentrations were determined by Bradford assay using Protein Assay Dye Reagent (BioRad, Mississauga, ON). Thirty micrograms of lysates were electrophoresed on an 8% sodium dodecyl sulphate-polyacrylamide electrophoresis gel and transferred to a polyvinylidene difluoride membrane (Roche, Mississauga, ON). Blots were blocked with 5% skim milk in Tris-buffered saline with Tween 20 (TBST; 10 mM Tris-HCl, pH 8.0, 150 mM NaCl, 0.1% Tween 20) for 1 h, then blots were incubated overnight on a rocking platform at 4 °C with specific antibodies at 1:1000 dilution in BSA/ TBST [anti-LDLR (Abcam, ab14056; Cambridge, MA); anti-actin (Sigma)]. Blots were washed using TBST and incubated with peroxidase- conjugated anti-rabbit IgG (GE Healthcare) for 1 h at 1:10,000 dilution in 5% skim milk/TBST for the α-LDLR antibody, or 5% BSA/TBST for α-actin at room temperature. Blots were washed again using TBST followed by incubation with Luminata Forte Western horseradish peroxidase substrate (Millipore, Etobicoke, ON) and visualized with the ChemiDoc MP System (BioRad, Mississauga, ON).

### Statistical analysis

Statistical significance was determined using GraphPad Prism 6 (GraphPad Software, San Diego, CA) by unpaired two-tailed Student’s *t*-test or one-way analysis of variance followed by a Tukey’s *posthoc* test, or Dunnett’s *posthoc* test when comparing to a single control sample. Levels of statistical significance indicated in each figure are as follows: *, *p* < 0.05; **, *p* < 0.01; ***, *p* < 0.001; ****, *p* < 0.0001.

## Results

### HGSC tumour cell heterogeneity impacts MRBV oncolytic efficacy

We commonly isolate cancer cells from the ascites of patients with metastatic EOC to perform in vitro cell culture experimentation [15]. From one EOC patient with a mixed HGSC and clear cell carcinoma (70% HGSC, 30% clear cell) and previously treated with debulking surgery and carboplatin/paclitaxel chemotherapy, we received four independent isolates over 14 months after disease recurrence (Fig. 1a). The resultant cell lines were confirmed to be HGSC with the universal presence of a *TP53* R280K missense mutation. Using these four lines we sought to investigate the effects of temporal changes on EOC cell susceptibility to MRBV oncolytic infection and cell killing. We observed that iOvCa105 and iOvCa147 cell lines, which were the first and last isolates received, were highly sensitive to MRBV with complete oncolysis achieved by MOI 0.01 after 3 d post-infection. This result was in stark contrast to iOvCa131 and iOvCa142 cell lines isolated from the same patient where only partial oncolysis was observed, even at MRBV concentrations as high as an MOI of 1 after 3 d of infection; complete oncolysis in these two lines was not achieved for any virus concentration tested (Fig. 1b). These results indicated that the metastatic HGSC cell population in this patient was dynamic over time and could be heterogeneous with respect to MRBV sensitivity.

**Fig. 2:**
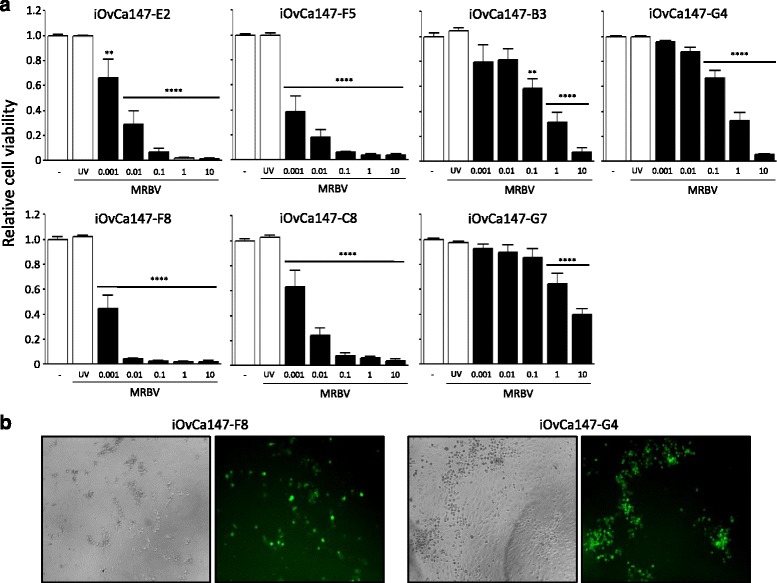
Intratumoral heterogeneity impacts MRBV oncolytic efficacy in vitro. **a**. Seven subcloned derivatives from iOvCa147 were generated through limiting dilution culturing and single-cell expansion to create homogeneous cell lines from the original heterogeneous cell line population. Resultant cells were seeded at 10,000 cells per well of a 96-well plate and infected 24 h post-seeding. Cells were assayed for viability using CellTiter-Glo at 72 h post-infection. (**, *p* < 0.01; ****, *p* < 0.0001, as determined by one-way ANOVA and Dunnett’s *posthoc* test). **b**. Images of MRBV-infected cells were captured at 72 h post-infection using bright-field and fluorescence microscopy at 50× original magnification

We next sought to explore this inherent intra-patient HGSC heterogeneity by using several subcloned lines expanded from single cells of the MRBV-sensitive iOvCa147 cell line. Using a set of seven different subclonal lines, MRBV infections were performed and viability was measured 3 d post-infection. We observed two distinct responses to MRBV infection among the clones: one group of four subclones demonstrated complete oncolysis at less than or equal to an MOI of 0.1, and another group of three subclones exhibited 1000-fold reduced sensitivity to MRBV (Fig. 2a). In fact, complete oncolysis was not achieved in these ‘resistant’ subclones even at an MOI of 10. Indeed, we observed only modest cytopathic effect (CPE) in small patches of GFP-positive iOvCa147-G4 resistant cellsafter 72 h of MRBV infection, whereas there was widespread CPE signifying productive MRBV replication in iOvCa147-F8 sensitive cells (Fig. 2b). Taken together, these results represent the first direct example of how both temporal and spatial heterogeneity in metastatic HGSC of the ovary can impact MRBV oncolytic efficacy.

### Low-density lipoprotein receptor (LDLR) is required for efficient MRBV entry

**Fig. 3:**
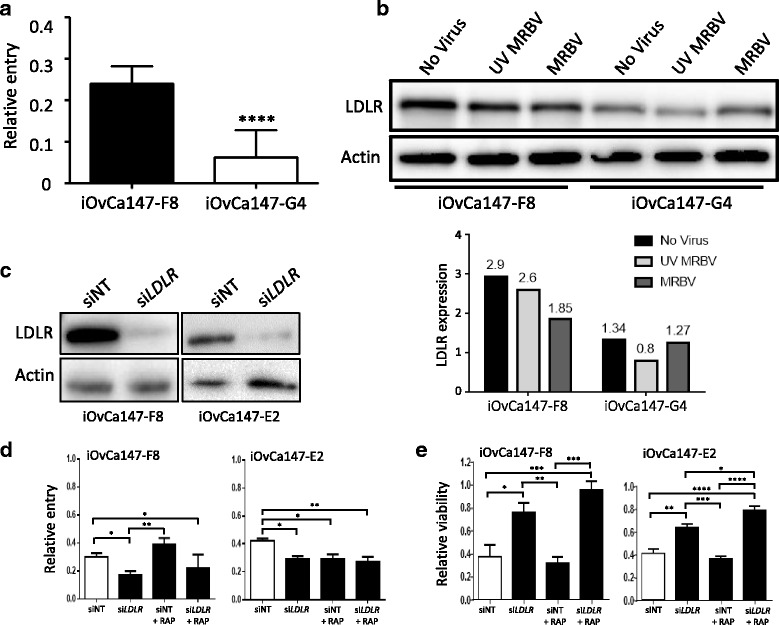
Differences in LDLR expression can impact MRBV entry. **a**. iOvCa147-F8 and iOvCa147-G4 cells were seeded at 75,000 cells per well of a 24-well plate. After 24 h, cells were infected with MRBV at an MOI of 1 for 1 h. Supernatant was collected and non-cell associated virus was titrated using Vero cells; supernatant from infections containing no cells were used as a control for total uninfected virus. **b**. Western blot were performed using lysates collected from iOvCa147-F8 and iOvCa147-G4 cells infected with MRBV at an MOI of 1, or UV-inactivated MRBV and no virus as controls. Actin served as a loading control. The graph represents quantification of LDLR expression performed using Bio-Rad Image Lab software and normalized to actin. **c**. Two different MRBV-sensitive subclones iOvCa147-F8 and iOvCa147-E2 cells were seeded at 20,000 cells per well of a 48-well plate. Cells were transfected with siNT or si*LDLR* and 48 h post-transfection, cells were harvested for protein lysates. Western blot for LDLR was performed and actin was used as a loading control. **d**. iOvCa147- F8 and iOvCa147-E2 cells transfected with siNT or si*LDLR* were treated with 100 nM of RAP for 1 h at 48 h post-transfection followed by MRBV infection for another 1 h. media was collected for titration of MRBV virus on Vero cells via plaque assay; treatments containing no cells were performed for normalization. **e**. iOvCa147- F8 and iOvCa147-E2 cells transfected with siNT or si*LDLR* were treated with 100 nM of RAP for 1 h at 48 h post-transfection followed by MRBV infection for another 1 h. Cells were assayed for viability using CellTiter-Glo at 72 h post-infection. (*, *p* < 0.05; **, *p* < 0.01; ***, *p* < 0.001; ****, *p* < 0.0001, as determined by one-way ANOVA and Tukey’s *posthoc* test)

As a first step to determine factors affecting differential MRBV oncolysis in HGSC cell subpopulations, we evaluated whether cell-associated MRBV was altered between sensitive and resistant subclone cell lines. We infected iOvCa147-F8 (sensitive) and iOvCa147-G4 (resistant) cell lines with MRBV for one hour after which we quantified the remaining virus in the media. Nearly 25% of MRBV entered iOvCa147-F8 cells, yet only 5% of MRBV entered iOvCa147-G4 cells (Fig. 3a).

We have previously established a link between the expression of LDLR and MRBV entry [10], therefore we determined whether differences in LDLR expression between the iOvCa147-F8 and iOvCa147-G4 subclones affects their susceptibility to MRBV infection. Analysis of gene copy-number alterations in serous ovarian adenocarcinoma using The Cancer Genome Atlas data [16, 17] revealed that over 10% of tumours show *LDLR* gene amplification with an additional 8/590 samples showing elevated mRNA expression (Additional file 1: Figure S1). Therefore, we determined whether LDLR protein was differentially expressed between sensitive and resistant clonal lines. We examined LDLR expression in iOvCa147-F8 and iOvCa147-G4 cells with and without MRBV infection. Indeed, iOvCa147-F8 cells expressed higher levels of LDLR as compared with iOvCa147-G4 cells (Fig. 3b). In response to both UV-inactivated MRBV (binds and enters cells, but does not replicate) and replication-competent MRBV, LDLR expression was slightly decreased in iOvCa147-F8 cells. This may indicate virus-binding to the LDLR, followed by endocytosis and lysosomal degradation of the internalized receptor [18]. This result was distinct from iOvCa147-G4 cells as the lower LDLR expression in this resistant subclone cell line did not change in response to virus (Fig. 3b), thus explaining deficiency in MRBV binding and entry.

We previously reported reduced LDLR expression in EOC cell lines during spheroid formation which correlated with decreased MRBV entry into spheroid cells, and this was confirmed by siRNA-mediated knockdown of LDLR expression in adherent cells [10]. Indeed, we observed a decrease in endogenous LDLR expression in iOvCa147-F8 spheroids, which correlated with statistically significant increase in cell viability after MRBV infection to levels equalling the resistant iOvCa147-G4 subclone (Additional file 2: Figure S2). With this correlative data linking LDLR expression with virus infectivity in iOvCa147-F8 and -G4 cells, we sought to determine if decreased LDLR would impact susceptibility of sensitive HGSC cells to virus infection. As expected, *LDLR* knockdown using SMARTpool siRNA in two different MRBV-sensitive subclone cell lines, iOvCa147-F8 and -E2 (Fig. 3c), significantly decreased MRBV entry (Fig. 3d) and oncolytic potential (Fig. 3E, and Additional file 3: Figure S3). Validation of *LDLR* silencing and ablation of MRBV oncolytic potential was performed using two individual siRNAs from the SMARTpool (Additional file 3: Figure S3a,b). The inhibitor of LDL-related receptors, RAP, reduced MRBV entry in iOvCa147-E2 clones, but not iOvCa147-F8 cells (Fig. 3d) and RAP had no effect on resultant MRBV oncolytic potential in either of these two sensitive HGSC subclone cell lines (Fig. 3e). These data indicate that LDLR is an important mediator controlling virus entry rather than its receptor family members similar to what we had documented previously [10].

Given that EOC cells require sufficient LDLR expression to mediate efficient MRBV entry [10], we sought to determine whether endogenous LDLR protein expression on its own was sufficient to predict HGSC cell susceptibility to MRBV oncolysis. We assessed LDLR expression in the four independent isolates, iOvCa105, iOvCa131, iOvCa142, and iOvCa147, and all seven subclone cell lines generated from iOvCa147. We did not observe, however, a direct correlation between sensitivity and resistance to MRBV infection with overall LDLR expression among all cell lines (Additional file 4: Figure S4). This suggests that although LDLR regulates MRBV entry into HGSC cells, its expression alone does not predict sensitivity to MRBV oncolytic infection.

### Secreted factors do not impart MRBV sensitivity or resistance

**Fig. 4:**
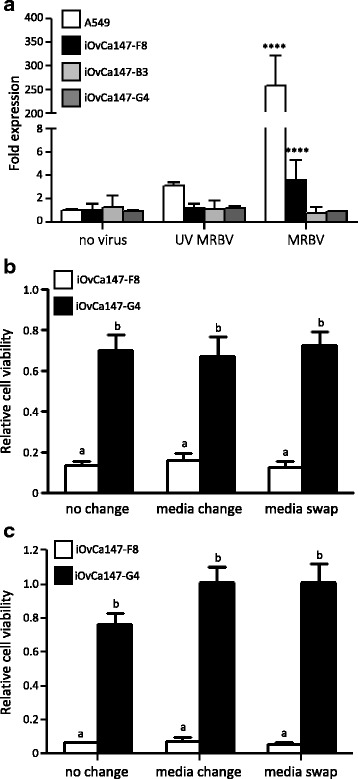
Extracellular factors do not impart sensitivity to MRBV. **a** MRBV-sensitive iOvCa147-F8, and MRBV-resistant iOvCa147-B3 and iOvCa147-G4 cells were seeded at 500,000 cells per well of a 6-well plate. A549 human lung adenocarcinoma cells served as a type I IFN response positive control cell line. The following day, cells were infected with MRBV at an MOI of 1 or UV-inactivated MRBV for 6 h. RNA was isolated to perform qRT-PCR using human-specific *IFNB1* primers and SYBR Green detection; *GAPDH* served as a normalization control. (****, *p* < 0.0001, as determined by one-way ANOVA and Dunnett’s *posthoc* test). **b** Cells were seeded at 10,000 cells per well of a 96-well plate. After 16 h, fresh media was either (i) replaced, (ii) conditioned media was swapped, or (iii) left unchanged between iOvCa147-F8 and iOvCa147-G4 cells. Cells were then immediately infected with MRBV at an MOI of 0.1 for 1 h followed by media change. CellTiter-Glo® assays were performed for cell viability 48 h post-infection. **c** iOvCa147-F8 and iOvCa-G4 cells were seeded at 10,000 cells per well of a 96-well plate. After 16 h, cells were infected with MRBV at an MOI of 0.1 for 1 h followed by media change. At 12 h, fresh media was either (i) replaced, or (ii) conditioned media was swapped between iOvCa147-F8 and iOvCa-G4 cells, or (iii) left unchanged. CellTiter-Glo® assays were performed for cell viability 48 h post-infection. (Letters indicate whether there is a statistically significant difference (*p* < 0.05) among conditions as determined by one-way ANOVA and Tukey’s *posthoc* test)

We next sought to determine whether MRBV-resistance in HGSC subclones is mediated by secreted factors that could reduce infection of sensitive cells. Since MRBV infection can elicit a robust anti-viral type I IFN response in normal cells [19], we first tested whether MRBV-infected HGSC cells generated this classic response. Quantitative RT-PCR analysis of *IFNB1* mRNA expression in sensitive iOvCa147-F8 cells, and two resistant subclones, iOvCa147-G4 and -B3 was performed after MRBV infection. Neither MRBV-sensitive nor -resistant HGSC subclone cell lines elicited a potent IFN antiviral signaling response after MRBV infection (Fig. 4a). The A549 human lung adenocarcinoma cell line, which is known to elicit a robust type I interferon response to virus infection with rapid upregulation of the *IFNB1* gene and thus serves as a positive control [13], responded to MRBV infection with a robust increase in *IFNB1* expression.

To further determine whether other secreted factors elicited an effect on sensitivity to MRBV, iOvCa147-F8 and -G4 cell lines were treated with conditioned media from the reciprocal subclone cell line (i.e., media swap) immediately prior to MRBV infection. Forty-eight hours after infection, we observed that the conditioned media from the reciprocal cell line did not alter sensitivity or resistance to MRBV oncolytic infection (Fig. 4b). Extracellular factors other than *IFNB1* produced after an acute MRBV infection may affect HGSC cell sensitivity or resistance; thus we performed another conditioned media swapping experiment, but using media shortly following MRBV infection. Again, there were no differences in cell viability when conditioned media from acutely-infected cells were swapped, indicating that secreted resistance factors are not transferred between iOvCa147-F8 (sensitive) and -G4 (resistance) subclones (Fig. 4c).

### Direct contact in MRBV-sensitive and -resistant cell co-cultures restores oncolysis

**Fig. 5:**
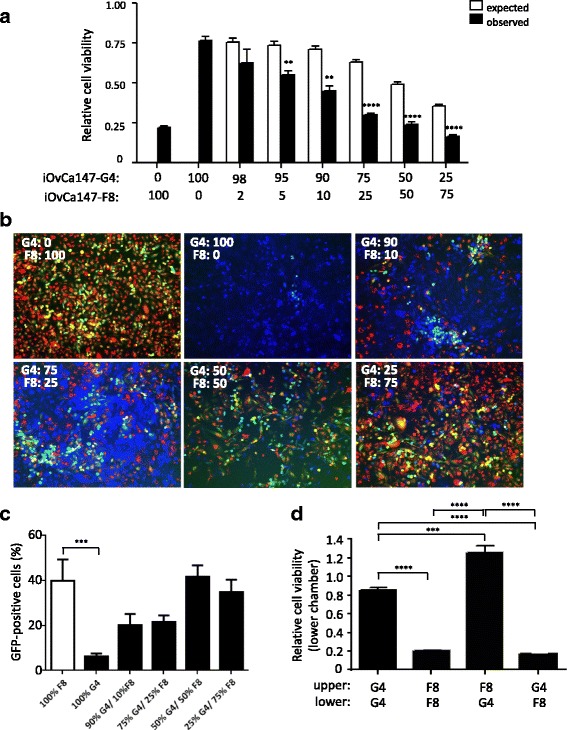
MRBV sensitivity can be conferred to resistant cells through direct cell-cell co-culture. **a** iOvCa147-F8 and iOvCa147-G4 cells were seeded at specific mixtures as indicated to a total of 100,000 cells per well of a 24-well plate. No virus mock-infections at each cell mixture was used as a control to determine relative viability as assessed at 48 h post-infection using CellTiter-Glo®. Expected viability if there was no interaction between subclones was calculated using the data from 100% pure iOvCa147-F8 and 100% pure iOvCa147-G4 MRBV-infected cultures. (**, *p* < 0.01; ****, *p* < 0.0001, as determined by paired Student’s *t*-test) **b** Fluorescence images of co-cultured cells iOvCa147-F8 (red, DiI-labelled) and iOvCa147-G4 (*blue*, CMAC-labelled) were captured 16 h post-infection. MRBV-infected iOvCa147-F8 cells appear *yellow* (GFP and DiI double-positive) whereas MRBV-infected iOvCa147-G4 cells appear teal (GFP and CMAC double-positive). **c** Double-positive cells were counted for each co-culture concentration and normalized to the total number of iOvCa147-G4 cells (*black bars*) to determine percent infectivity (100% iOvCa147-F8 served as a control; white bar). (***, *p* < 0.001, as determined by one-way ANOVA and Dunnett’s *posthoc* test) **d** Physical separation of cells was achieved using 0.4-μm Transwell inserts. Media was identical between both upper and lower chambers and 100,000 cells in total were seeded (25,000 cells in the upper chamber and 75,000 cells in the lower chamber). After 24 h, media was changed and cells were infected at 50,000 viral particles (MOI 0.5) of MRBV. After 48 h, viability of the cells in the lower chamber only was measured using CellTiter-Glo® and normalized to uninfected cells. (***, *p* < 0.001; ****, *p* < 0.0001, as determined by one-way ANOVA and Tukey’s *posthoc* test)

Lastly, we sought to determine whether direct interaction of MRBV-sensitive and –resistant cells within a heterogeneous tumour cell population might impact oncolytic efficacy. We predicted that efficient MRBV infection and oncolysis would be restored in this context since the original iOvCa147 mixed population cell line was quite sensitive to MRBV oncolytic infection (Fig. 1b). To recapitulate various iterations of a heterogeneous tumour population, we co-cultured iOvCa147-F8 and iOvCa147-G4 cells at multiple different ratios. Indeed, MRBV-mediated cell killing was increased at each co-culture ratio than what would be expected for each individual subclone if targeted independently by MRBV (Fig. 5a). In fact, the co-culture having an equal ratio of both sensitive iOvCa147-F8 and resistant iOvCa147-G4 cells completely restored MRBV oncolytic potential.

To directly visualize MRBV infection into each subclonal cell line within mixed co-cultures, we pre-labelled cells with cell-permeable fluorescence dyes. Individual fluorescence labeling was achieved in iOvCa147-F8 cells using DiI and in iOvCa147-G4 cells using CMAC prior to co-culture. Infection was then visualized using the MRBV-driven expression of GFP. We observed enhanced MRBV infection of iOvCa147-G4 cells by the increased proportion of CMAC and GFP double-positive cells when co-cultured with infected iOvCa147-F8 cells (DiI and GFP double positive) (Fig. 5b). Indeed, co-cultures achieved a 9-fold increase in MRBV-infected iOvCa147-G4 cells when compared to MRBV infection of iOvCa147-G4 cells alone (Fig. 5c). Finally, the requirement of direct cell-cell contact in these co-cultures for efficient MRBV infection was validated by physically separating sensitive iOvCa147-F8 and resistant iOvCa147-G4 subclone cell lines using a porous Transwell membrane during active infection. As expected, this physical separation of cell lines abrogated the re-sensitization of iOvCa147-G4 cells to MRBV infection (Fig. 5d). Overall, our findings support MRBV as an effective therapeutic agent to infect and kill HGSC cells throughout a heterogeneous tumour, as long as there are subpopulations of sensitive cells to perpetuate complete oncolysis.

## Discussion

There continues to be a dire and unmet need for novel therapeutic alternatives for the treatment of metastatic EOC due to the high rate of chemo-resistance in recurrent disease [20]. This is due in large part to complexity in histological subtypes of this EOC, as well as substantial genomic instability in high-grade disease that can drive tumour heterogeneity. We previously showed the oncolytic agent MRBV to be a potent therapeutic agent in EOC cells in cultured cells and spheroids, thus we sought to evaluate MRBV oncolytic efficiency in the context of tumour heterogeneity. Using independently isolated EOC cell lines derived directly from a patient during her relapsed disease course, we observed differential oncolytic efficacies for MRBV. Likewise, individual subclones generated from a MRBV-sensitive heterogeneous cell line isolate actually consisted of a mixture of both sensitive and resistant subpopulations. These findings are the first to highlight the potential impact that dynamic intratumoral heterogeneity can have on therapeutic efficacy. Our data represents the first evidence for both temporal and spatial heterogeneity in using oncolytic virus therapeutics for EOC.

Inherent cancer cell pathobiology and its response to treatment can act as strong selective pressures to change patterns in EOC growth, spread and ultimately therapy-resistant disease recurrence [21]. Since debulking surgery and ascites alleviation are imperative to the clinical management of metastatic EOC [1, 2], this affords a unique opportunity to evaluate potential impact of dynamic changes in ovarian tumour biology on therapeutic efficacy and resistance mechanisms. The selective pressure that would act upon the ovarian tumour cells present in each isolate of ascites results from cytotoxic chemotherapy administration in the patient. Indeed, over time the patient in our study developed resistance to standard carboplatin and paclitaxel combination chemotherapy as indicated in Fig. 1a. Since our in vitro studies used MRBV as a single biologic anti-tumour agent with a vastly different mechanism of action from chemotherapy, it is unlikely that the tumour cells would naturally evolve resistance to MRBV without being exposed to this selective pressure during the clinical course of the disease.

An obvious limitation of extended culture of cell lines is the accumulation of novel behaviours that do not necessarily reflect the original primary cell population. To minimize this potential cell culture artifact in our studies, cell lines and subclones were cultured for a restricted period during experimentation to limit the acquisition of additional mutations via selective pressure. In addition, preliminary OncoPanel™ testing did not identify any novel gene mutation acquisition among the analyzed cell lines, at least for the subset of hotspot mutations available using this platform (Ramos Valdes & DiMattia, unpublished data). Most importantly, we identified that four independent subclones retained sensitivity to MRBV-mediated oncolysis, whereas three other subclones exhibited a profound and reproducible MRBV-resistant phenotype. Acquisition of specific resistance to such an agent due to random genetic drift in cell culture without any selective pressure would be highly unlikely to occur with such regularity. In fact, we made the direct observation that among four different ascites samples collected over time from a single patient existed changing proportions of cells with differing sensitivity to MRBV infection and cell killing. Other evidence of this potential flux in clonal evolution has been recently documented in high-grade serous ovarian cancer, a highly-genomically unstable and aggressive histotype of this disease [6, 22]. It should be noted that although the pathology of the primary tumour from the patient in our study was of mixed high-grade serous (70%) and clear cell (30%) histologies, we had confirmed the consistent presence of a single *TP53* missense mutation in every cell line. *TP53* mutations are regarded as universal in ovarian HGSC [4]; but we saw no evidence of any mutations commonly seen in clear cell carcinomas of the ovary [23].

Cell surface receptor expression can act as a key control point for oncolytic virus specific targeting of cancer cells. Previously, we demonstrated that LDLR expression is an important factor mediating MRBV entry and efficient oncolysis of several established EOC cell lines [10]. Herein, we confirmed that LDLR, but not its LDLR-related receptors, is required for MRBV binding and entry in new patient-derived heterogeneous EOC cell lines also. However, we were unable to show that differences in LDLR expression level among sensitive and resistant subclones is sufficient to dictate MRBV infectivity. This does not rule out the possibility that other trophic factors may act in concert with LDLR directly or indirectly to define sensitivity to MRBV infection among cell subpopulations in EOC. From a virus entry standpoint, rhabdoviruses utilize phospholipids, gangliosides and protein receptors, and these general mechanisms may serve as points of entry for MRBV as well [24, 25]. Alternatively, other extrinsic factors may impact oncolytic virus anti-tumour efficacy. For instance, Ilkow and colleagues reported recently that VSV-mediated cell killing can be modulated by both TGFβ and FGF2 cytokines by blocking type I interferon anti-viral responses [26]. This study showed that secreted factors from the cancer-associated microenvironment were implicated in affecting oncolytic virus infection. Our results were focused on the malignant cellular component of these HGSC samples, and we demonstrated that conditioned media from EOC cells either before or after infection did not contain secreted factors conferring sensitivity or resistance to MRBV-mediated oncolysis. Understanding the full repertoire of both exogenous and endogenous trophic factors utilized by MRBV is critical to better target heterogeneous tumour cells and their associated microenvironment while minimizing off-target effects at other normal tissue sites.

Our results demonstrate conclusively that direct cell-cell contact of sensitive and resistant cell populations is required for efficient total MRBV infection. As such, we speculate that the formation of a virological synapse allows direct cell-cell transmission of MRBV from sensitive cells to infect resistant cells. This occurs when virus assembly components polarize at the site of the synaptic junction allowing for direct transfer of mature virus to an adjacent cell rather than having large excesses of cell-free virus [27, 28]. For example, vesicular stomatitis virus (VSV), can spread directly between cells via these synapses [29]. In fact, intravenously injected VSV is rarely observed as cell-free particles in vivo and are normally rapidly sequestered by cells either by infection or adhesion; surface attachment of VSV has been shown to increase infectivity by augmenting viral passage between cells [30]. Taken together, our results directly support the premise that MRBV, which is a VSV-related rhabdovirus, is transmitted via direct cell-cell contact, and specifically to facilitate MRBV infection from sensitive to resistant cells. These findings imply that even in a heterogeneous ovarian tumour only a small subpopulation of cells needs to be efficiently infected to result in complete oncolytic infection of the tumour. Our results also support the strategy to use cell-based carrier systems for MRBV delivery, especially to enhance infectivity of the potentially resistant subpopulation cells in a heterogeneous tumour [31].

## Conclusions

One postulation is that cancer therapy resistance can arise from the expansion of inherently resistant subpopulations that are already present within a heterogeneous tumour. Indeed, we observed that within a mixed population of EOC cells from a single patient sample, there can reside both MRBV-sensitive and -resistant clones of cells even prior to exposure to virus. However, our results demonstrate that efficient MRBV infection of neighbouring sensitive clones can render resistant clones susceptible to oncolytic-mediated cell death, which may reduce the probability of future therapy resistance. Future studies would be important to directly assess whether selective forces imposed by MRBV treatment could eventually give rise to a dominant MRBV resistant population of cells from heterogeneous EOC, or if alternative adaptive responses are required to support resistance. Direct application of heterogeneous patient-derived tumour specimens in pre-clinical oncolytics research could uncover novel strategies to better exploit these anti-tumour viral agents, like MRBV, for broader therapeutic use in treating EOC at specific time points during disease progression and preventing development of resistance to oncolytic therapy.

## Additional files

Additional file 1: Figure S1. Highest alteration frequency of the *LDLR* gene in serous ovarian cancers. a. Mutation and gene copy-number status for *LDLR* across human cancers as determined using The Cancer Genome Atlas (TCGA) provisional datasets accessed from cBioPortal as of November 12, 2015. Ovarian cancer (serous adenocarcinoma) has the highest prevalence of *LDLR* gene alterations, particularly amplifications, as compared with all other malignancies in the data set. b. Oncoprint of serous ovarian tumours harbouring *LDLR* mutations, copy-number changes, and gene expression changes (z-score > 2) from the TCGA provisional dataset. Only tumour samples with alterations (13%; 78/599 samples) are displayed for clarity. c. *LDLR* mRNA expression as compared with gene copy-number status among all serous ovarian tumours from the TCGA provisional dataset. (PPTX 240 kb)

Additional file 2: Figure S2. Reduced LDLR expression in spheroids decreases MRBV-mediated oncolysis. a. Cells were seeded into standard tissue culture plates or ultra-low attachment (ULA) plates to form spheroids and harvested for protein lysis 24 h after seeding. Western blotting was performed for LDLR expression and actin served as a loading control. LDLR expression is reduced in iOvCa147-F8 spheroids to similar levels seen in iOvCa147-G4 cells and spheroids. b. iOvCa147-F8 and -G4 cells were seeded at 50,000 cells per well of a 24-well ULA plate and spheroids were formed over 72 h. Spheroids were then infected with MRBV at an MOI of 0.1 for 48 h and viability was assessed using CellTiter-Glo®; MRBV-infected adherent cells were used for comparison. (PPTX 65 kb)

Additional file 3: Figure S3. Validation of *LDLR* knockdown using two independent siRNAs. a. iOvCa147-F8 cells were seeded at 20,000 cells per well of a 48-well dish, then transfected with each si*LDLR* siRNA or siNT control for 48 h. Transfected cells were harvested for protein lysis to perform western blotting for LDLR expression. b. Cells transfected with si*LDLR-1*, si*LDLR-2*, or siNT, were infected with MRBV at an MOI of 0.05 for 48 h and viability was measured using CellTiter-Glo®. (PPTX 85 kb)

Additional file 4: Figure S4. LDLR protein expression in EOC patient-derived cell lines and subclones used in this study. Western blotting for LDLR protein expression was performed using lysates generated from the indicated cell lines and iOvCa147 subclones. Classification of cell line and subclone sensitivity or resistance to MRBV infection are indicated. (PPTX 63 kb)

## Abbreviations

CPE: Cytopathic effect
EOC: Epithelial ovarian cancer
GFP: Green fluorescent protein
HGSC: High-grade serous cancer
IFN: Interferon
LDLR: Low-density lipoprotein receptor
MOI: Multiplicity-of-infection
MRBV: Maraba virus
RAP: Receptor-associated protein
STR: Short-tandem repeat
ULA: Ultra-Low Attachment
VSV: Vesicular stomatitis virus
