# Supplementary figures and images for: Spatial and temporal epithelial ovarian cancer cell heterogeneity impacts Maraba virus oncolytic potential
Source: BMC Cancer. 2017 Aug 30;17:594. doi: 10.1186/s12885-017-3600-2 (PMC5577660; doi:10.1186/s12885-017-3600-2)

## Slide 1
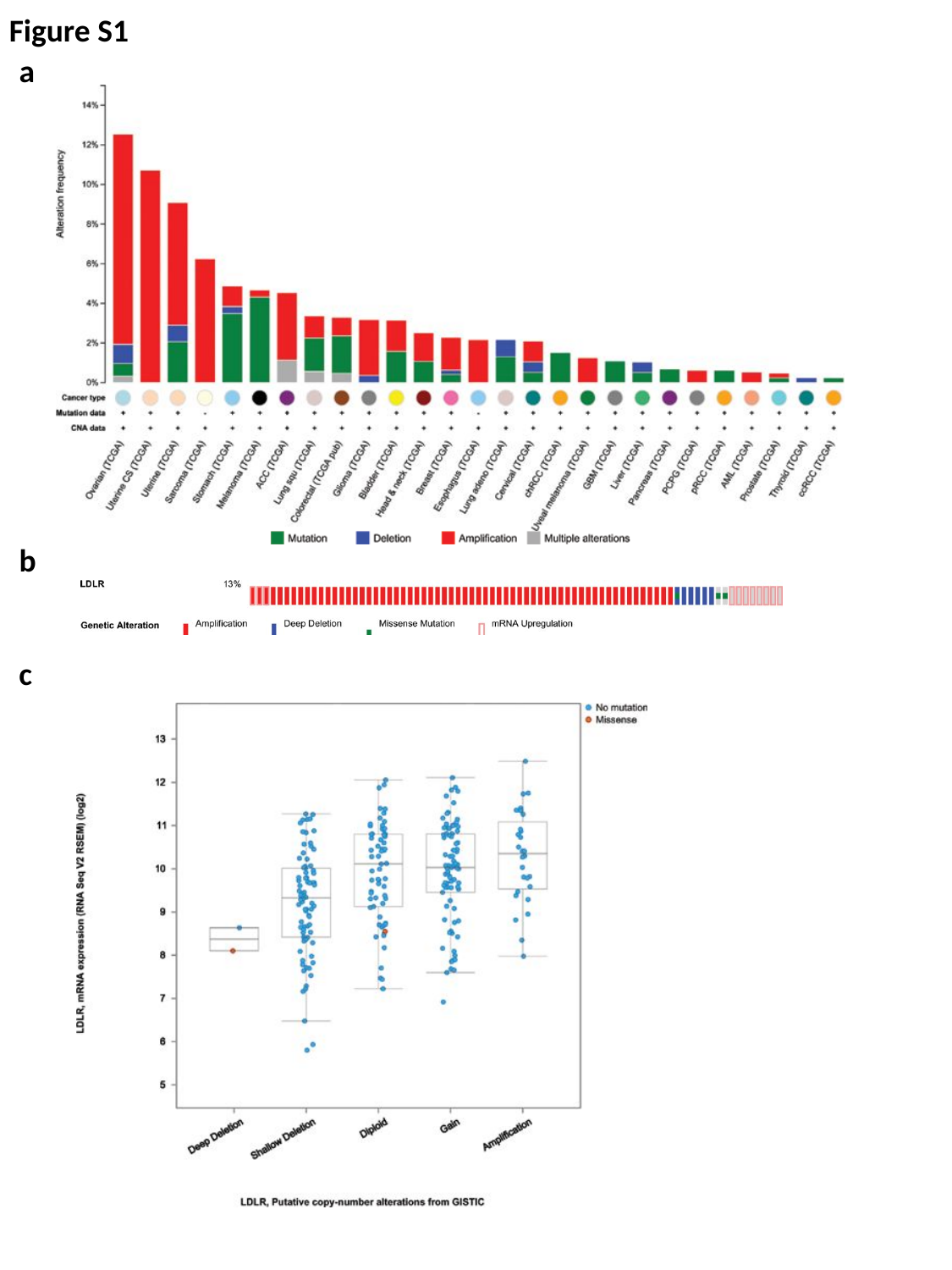

Figure S1
a
b
c

Supplement: Supplementary file 1 — Highest alteration frequency of the LDLR gene in serous ovarian cancers. a. Mutation and gene copy-number status for LDLR across human cancers as determined using The Cancer Genome Atlas (TCGA) provisional datasets accessed from cBioPortal as of November 12, 2015. Ovarian cancer (serous adenocarcinoma) has the highest prevalence of LDLR gene alterations, particularly amplifications, as compared with all other malignancies in the data set. b. Oncoprint of serous ovarian tumours harbouring LDLR mutations, copy-number changes, and gene expression changes (z-score > 2) from the TCGA provisional dataset. Only tumour samples with alterations (13%; 78/599 samples) are displayed for clarity. c. LDLR mRNA expression as compared with gene copy-number status among all serous ovarian tumours from the TCGA provisional dataset. (PPTX 240 kb) [file 12885_2017_3600_MOESM1_ESM.pptx]

## Slide 1
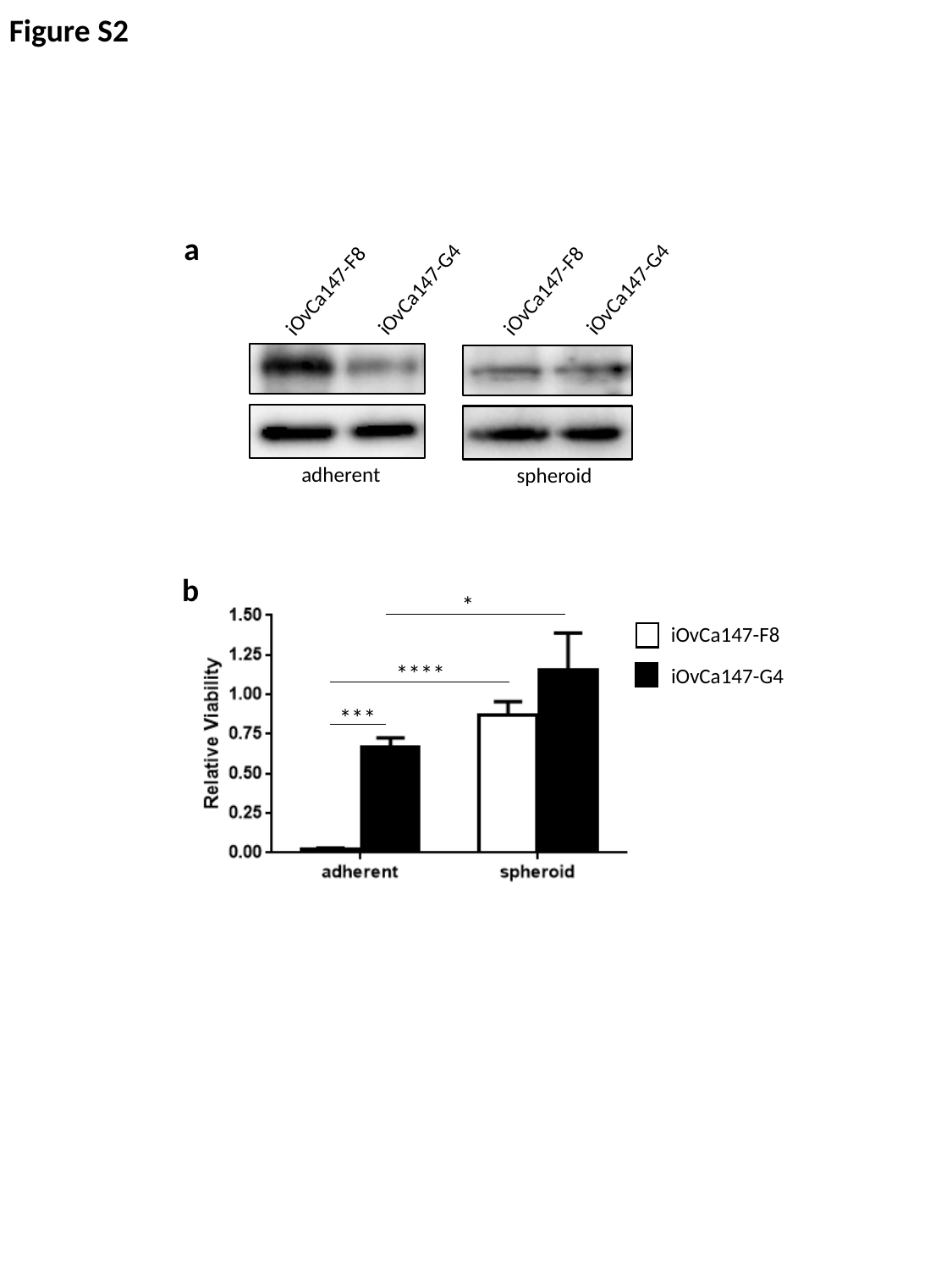

Figure S2
a
iOvCa147-G4
iOvCa147-G4
iOvCa147-F8
iOvCa147-F8
adherent
spheroid
b
*
iOvCa147-F8
****
iOvCa147-G4
***

Supplement: Supplementary file 2 — Reduced LDLR expression in spheroids decreases MRBV-mediated oncolysis. a. Cells were seeded into standard tissue culture plates or ultra-low attachment (ULA) plates to form spheroids and harvested for protein lysis 24 h after seeding. Western blotting was performed for LDLR expression and actin served as a loading control. LDLR expression is reduced in iOvCa147-F8 spheroids to similar levels seen in iOvCa147-G4 cells and spheroids. b. iOvCa147-F8 and -G4 cells were seeded at 50,000 cells per well of a 24-well ULA plate and spheroids were formed over 72 h. Spheroids were then infected with MRBV at an MOI of 0.1 for 48 h and viability was assessed using CellTiter-Glo®; MRBV-infected adherent cells were used for comparison. (PPTX 65 kb) [file 12885_2017_3600_MOESM2_ESM.pptx]

## Slide 1
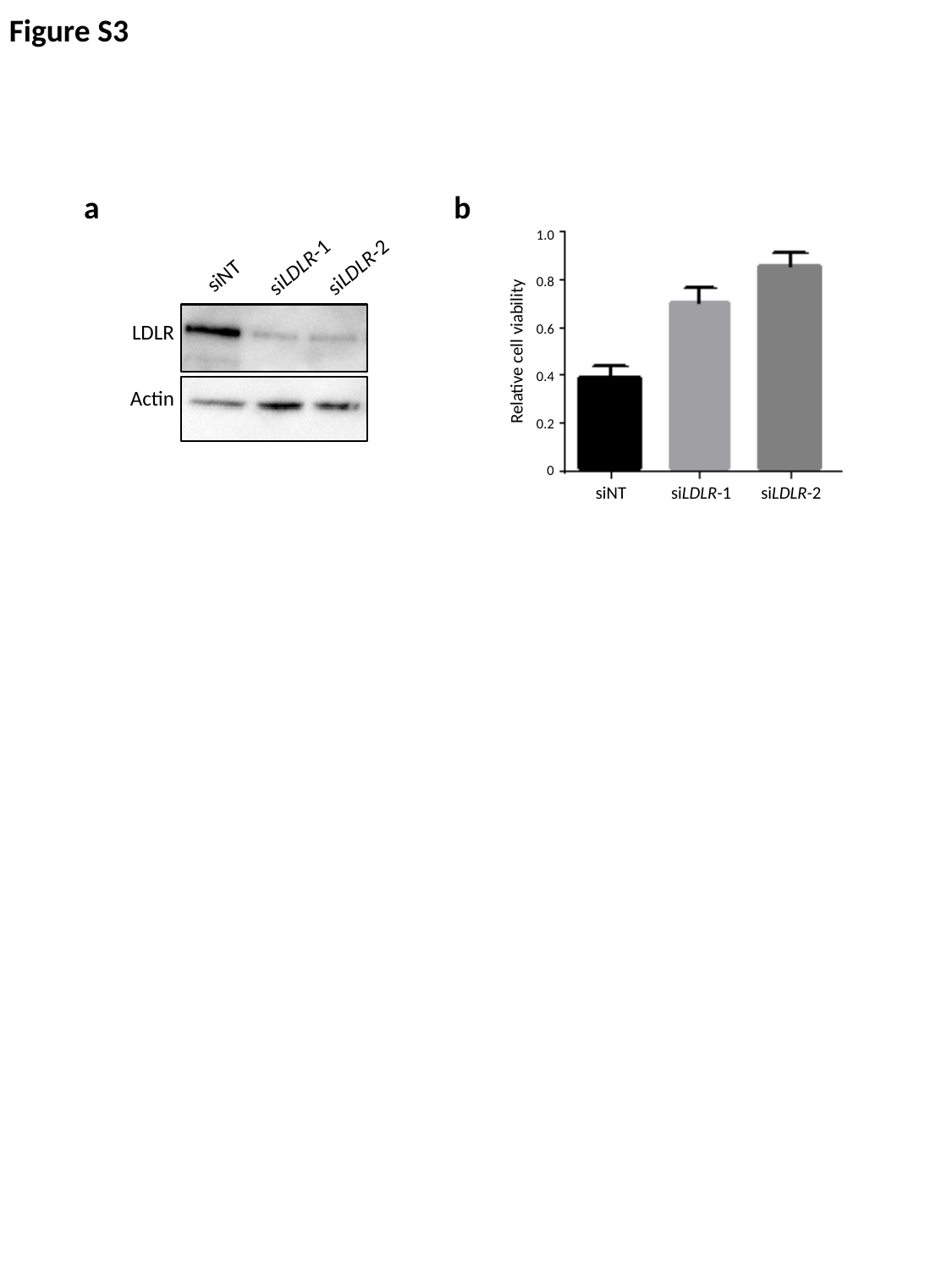

Figure S3
a
b
1.0
0.8
0.6
Relative cell viability
0.4
0.2
0
siNT
siLDLR-1
siLDLR-2
siLDLR-1
siLDLR-2
siNT
LDLR
Actin

Supplement: Supplementary file 3 — Validation of LDLR knockdown using two independent siRNAs. a. iOvCa147-F8 cells were seeded at 20,000 cells per well of a 48-well dish, then transfected with each siLDLR siRNA or siNT control for 48 h. Transfected cells were harvested for protein lysis to perform western blotting for LDLR expression. b. Cells transfected with siLDLR-1, siLDLR-2, or siNT, were infected with MRBV at an MOI of 0.05 for 48 h and viability was measured using CellTiter-Glo®. (PPTX 85 kb) [file 12885_2017_3600_MOESM3_ESM.pptx]
