# Supplementary material for: Spatial and temporal epithelial ovarian cancer cell heterogeneity impacts Maraba virus oncolytic potential
Source: BMC Cancer. 2017 Aug 30;17:594. doi: 10.1186/s12885-017-3600-2 (PMC5577660; doi:10.1186/s12885-017-3600-2)
Supplement: Supplementary file 4 — LDLR protein expression in EOC patient-derived cell lines and subclones used in this study. Western blotting for LDLR protein expression was performed using lysates generated from the indicated cell lines and iOvCa147 subclones. Classification of cell line and subclone sensitivity or resistance to MRBV infection are indicated. (PPTX 63 kb) [file 12885_2017_3600_MOESM4_ESM.pptx]

## Slide 1
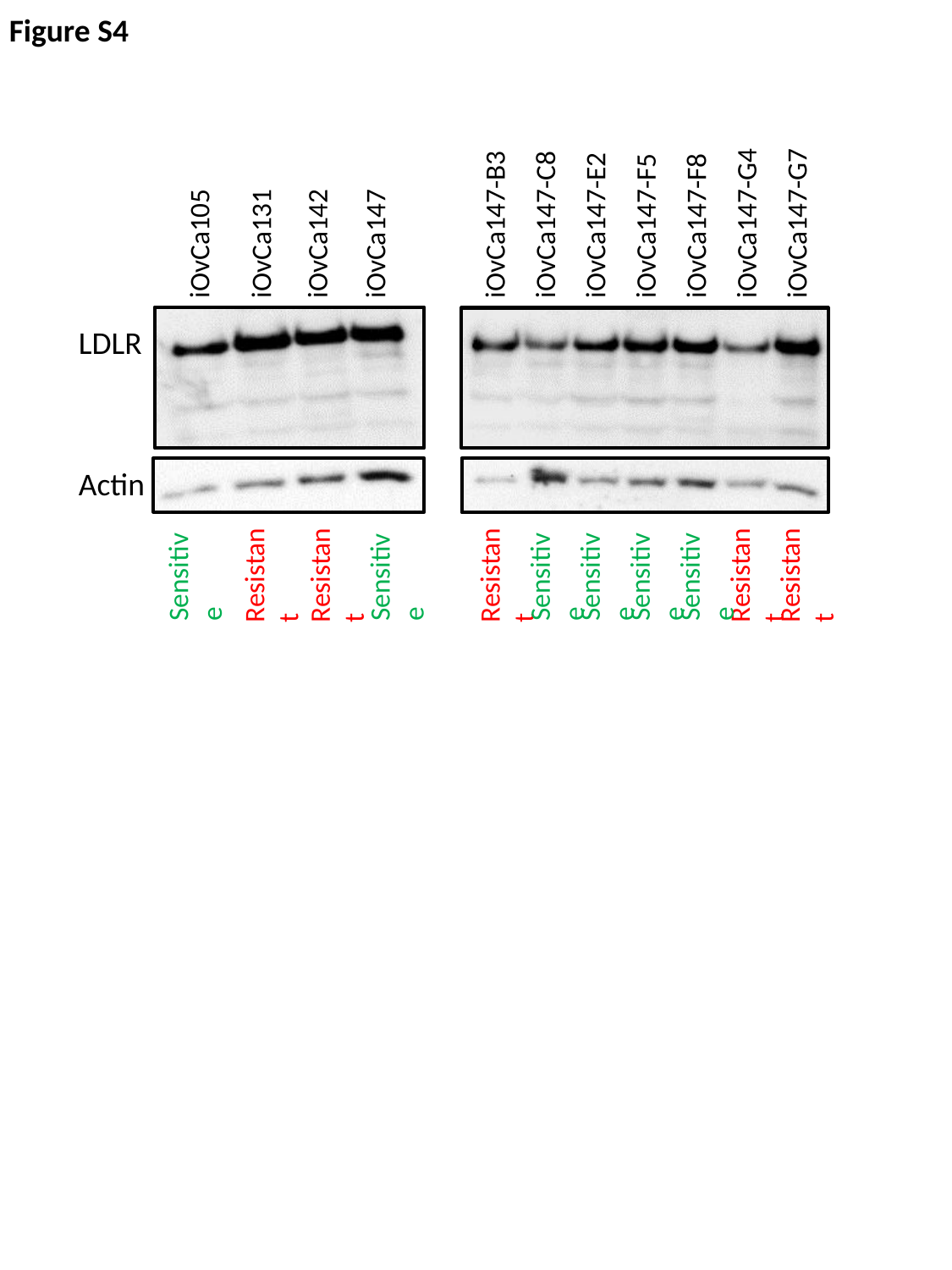

Figure S4
iOvCa105
iOvCa131
iOvCa142
iOvCa147
iOvCa147-B3
iOvCa147-C8
iOvCa147-E2
iOvCa147-F5
iOvCa147-F8
iOvCa147-G4
iOvCa147-G7
LDLR
Actin
Sensitive
Resistant
Resistant
Sensitive
Resistant
Sensitive
Sensitive
Sensitive
Sensitive
Resistant
Resistant
